# Presentation, Management, and Outcomes of Nonfunctioning Pituitary Adenomas: An Experience from a Developing Country

**DOI:** 10.7759/cureus.5759

**Published:** 2019-09-25

**Authors:** Bhagwan Das, Sumera Batool, Adeel Khoja, Najmul Islam

**Affiliations:** 1 Endocrinology, Aga Khan University Hospital, Karachi, PAK; 2 Endocrinology, Diabetes and Metabolism, Aga Khan University Hospital, Karachi, PAK; 3 Medicine, Aga Khan University Hospital, Karachi, PAK

**Keywords:** pituitary adenoma, surgery, radiotherapy

## Abstract

Objective

The goal of this study was to evaluate the presentation, management, and clinical outcome of nonfunctioning pituitary adenomas (NFPAs) in a tertiary care setup.

Methods

We conducted a retrospective review of patient records of 157 patients with the diagnosis of NFPA managed at Aga Khan University Hospital, a tertiary care hospital in Karachi, Pakistan from January 1, 2007, to December 31, 2017. We collected data on basic demographic characteristics, signs, and presenting symptoms, management, and outcomes. Data analysis was performed by using Stata, Version 12 (StataCorp LLC, College Station, TX).

Results

Most patients in the study were men (59%), and the mean age of the study population was 48 ± 14 years. The main presentations of NFPA were visual disturbance (77%) and headache (55%). In 78% of patients, the tumor was >1 cm on MRI. Most (87%) of patients underwent surgical resection, and of those, 93% received transsphenoidal surgery that was well tolerated. In the study population, 31% of patients had transient, 9% had permanent diabetes insipidus, and 25% developed hyponatremia. Of those in the study, 53% had low cortisol, 57% had hypothyroidism, and 27% needed sex hormone replacement after surgery. Residual tumor was confirmed in 43% of patients by postoperative MRI. Tumor recurrence and regrowth occurred in 17 patients and required repeat resection or radiosurgery.

Conclusion

In Pakistan, patients with NFPAs are more likely to present during the later stage, with larger adenoma and compressive symptoms compared to patients in developed countries. For the detection of residual disease and tumor recurrence, close screening and a multidisciplinary approach are needed after surgery.

## Introduction

Adenomas of the pituitary gland are mostly benign neoplasms constituting 5% to 20% of all intracranial tumors [1]. The annual estimated incidence rate of pituitary adenoma is approximately 20 cases per 100,000 population, making these adenomas the third most common among primary intracranial tumors after gliomas and meningiomas [2].

Nonfunctioning pituitary adenomas (NFPAs) account for approximately 15% to 30% of all adenomas of the pituitary gland [3,4]. NFPAs are characterized by the absence of biochemical and clinical evidence of pituitary hormonal overproduction [5]. The clinical presentation of NFPAs varies from completely asymptomatic to symptoms caused by the mass effect by the tumor resulting in a headache, visual disturbances, and pituitary apoplexy or with symptoms pituitary hormone deficiency. 

Transsphenoidal surgery (TSS) is recommended as the first-line treatment for patients with symptomatic NFPAs, especially in patients with visual field defects, as it leads to long-term tumor control in approximately 80% to 90% of patients and carries a low rate of mortality when performed by skilled surgeons [6-8]. Tumor resection rate after TSS is variable from 20% to 83%; approximately 50% of patients retain residual disease after surgery and need repeat surgery or radiation to decrease residual disease size, tumor regrowth, and recurrence [9].

The data regarding NFPAs in our population are inconsistent, and, to the best of our knowledge, there are no local data on the mode of presentation, endocrinal status, and management outcomes. The current study aims to evaluate the presentation, management, and outcomes of patients with NFPAs who presented to a tertiary care hospital in Pakistan.

## Materials and methods

This was a retrospective study conducted in Aga Khan University Hospital Karachi, one of the largest tertiary care hospitals in Pakistan. We reviewed the medical records of patients who were diagnosed with NFPAs and underwent surgery for pituitary adenoma at our hospital from January 1, 2007, to December 31, 2017. The study protocol was approved by the ethics review committee of the university (ERC number 5379-Med-ERC-2018). To preserve confidentiality, we coded each patient and removed their original identification. Data were collected by the primary author and noted in a predefined questionnaire.

We started the file review from the most recent case and recorded the demographic data, including age and gender. We then evaluated medical records and collected data on the presenting concerns, preoperative MRI and biochemical workup, clinical indications for surgery, postoperative hemodynamic status, and hormonal deficiencies. For the outcomes, we reviewed inpatient and outpatient notes, presurgery and postsurgery laboratory results, and scans of patients’ follow-up at our hospital after their surgical procedures.

To be included in our study, patients had to be 18 years old or older, of either sex, with radiologically reported pituitary adenomas in the absence of any clinical and biochemical signs of hormone excess. We excluded all patients with the diagnosis of pituitary adenomas and with any of biochemical activity on hormonal workup.

Statistical analysis

Mean with standard deviation was reported for symmetrically distributed continuous variables while median with interquartile range was reported for asymmetrically distributed continuous variables. The normality of the continuous variable was assessed through histogram by plotting a normal density plot on the graph. Frequencies with percentages were reported for all categorical variables. All analyses were performed on Stata, Version 12 (StataCorp LLC, College Station, TX).

## Results

**Figure 1:**
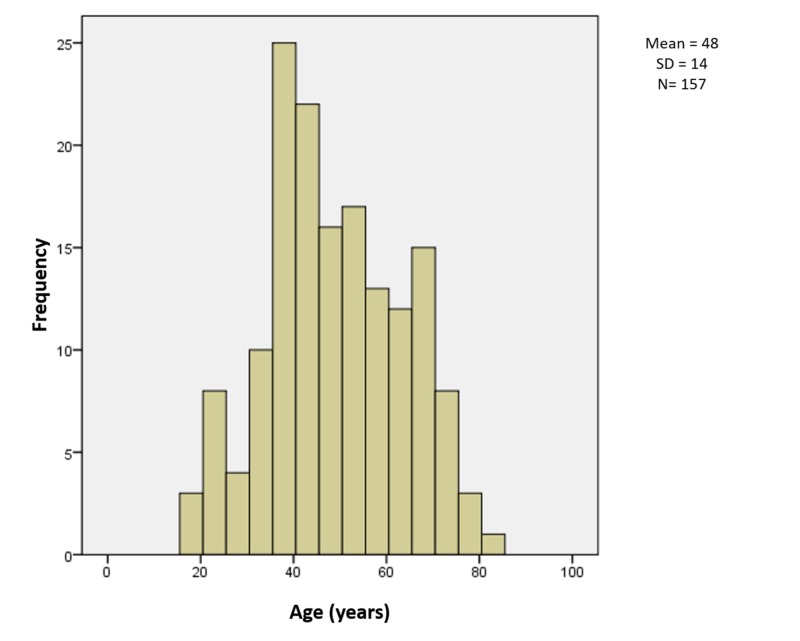
Histogram for variable of age

The mean age of study participants with NFPAs was 48 ± 14 years (Figure 1). Most participants (59%) were men, and 55% presented with headache and 77% presented with visual disturbances. Several patients (11%) presented with symptoms of hypothyroid, while 10% were diagnosed with pituitary apoplexy, and 6% had symptoms of hypocortisolism. A small portion of the study population (1%) had symptoms of growth hormone deficiency, and 6% had symptoms of gonadotrophic deficiency.

On imaging, 78% of our total study population had macroadenoma (> 1.0 cm). Preoperative replacement of steroid, thyroxine, growth hormone and sex hormone among patients with nonpituitary adenomas was required in 17%, 29%, 1%, and 3%, respectively. A total of 137 (87%) study participants underwent surgical management for their pituitary adenomas. Among them, 127 (93%) underwent TSS while 10 (7%) had transcranial surgery-a decision based on the large size of the adenoma and consultation with the surgeon. Of 121 study participants who had visual disturbances, 83 (69%) had an improvement in their visual disturbances.

Most patients who underwent surgical management (n = 137; 87%) had some postoperative complications. Among them, 55 study participants (40%) had diabetes insipidus (DI; 31% had transient, and 9% had permanent), 34 (25%) had hyponatremia, 22 (16%) had leakage of cerebrospinal fluid, 17 (12%) had cranial nerve palsy, 73 (53%) had hypocortisolism, 78 (57%) had hypothyroidism, and 37 (27%) needed sex hormone replacement after surgery.

There were two postoperative mortalities within eight weeks of surgery as a result of pulmonary embolism. Among 137 participants who underwent surgery, 58 (43%) had a residual tumor. In addition to this, 77 study participants (57%) had no residual tumor, and 17 (22%) had tumor recurrence or regrowth. Of 75 cases who had a residual tumor and recurrence, 29 (39%) had their first repeat surgery while other patients refused further intervention. Among these 29 study participants, nine (31%) had their second repeat surgery. Of the 75 cases who had a residual tumor and recurrence, 13 received external radiation therapy (17%). Among 62 study participants who did not receive conventional radiation therapy, 10 (16%) underwent cyber/gamma knife procedure (Table 1).

**Table 1:** Baseline characteristics of non-functioning pituitary adenomas: presentation, management, and outcomes *Age variable is symmetrically distributed, hence we are reporting mean with standard deviation. Abbreviations: NFPA, nonfunctioning pituitary adenoma; CSF, cerebrospinal fluid.

S. No	Variables	Study participants with NFPAs (N =157) (%)
Presentation		
1.	Age (years)*	48 ±14
2.	Gender	Male = 93 (59%)
		Female = 64 (41%)
3.	Presenting with Headache	Yes = 86 (55%)
		No = 71 (45%)
4.	Presenting with Visual Disturbances	Yes = 121 (77%)
		No = 36 (23%)
5.	Pituitary Apoplexy	Yes = 16 (10%)
		No =141 (90%)
6.	Presenting with Symptoms of Hypothyroid	Yes = 17 (11%)
		No = 140 (89%)
7.	Presenting with Symptoms of Hypocortisolism	Yes = 9 (6%)
		No = 148 (94%)
8.	Presenting with Symptoms of Growth Hormone Deficiency	Yes = 2 (1%)
		No = 155 (99%)
9.	Presenting with Symptoms of Gonadotrophic Deficiency	Yes = 9 (6%)
		No = 148 (94%)
10.	Tumor Size on Imaging	Micro adenoma (< 1.0 cm) = 35 (22%)
		Macro Adenoma (> 1.0 cm) = 122 (78%)
11.	Steroid Replacement (Preoperative)	Yes = 26 (17%)
		No = 131 (83%)
12.	Thyroxine Replacement (Preoperative)	Yes = 46 (29%)
		No = 111 (71%)
13.	Growth Hormone Replacement (Preoperative)	Yes = 1 (1%)
		No = 156 (99%)
14.	Sex Hormone Replacement (Preoperative)	Yes = 5 (3%)
		No = 152 (97%)
Management		
15.	Surgical Management	Yes = 137 (87%)
		No = 20 (13%)
16.	Type of Surgery	Trans sphenoidal Surgery = 127 (93%)
		Trans cranial Surgery = 10 (7%)
Outcomes		
17.	Improvement in Visual Disturbances	Yes = 83 (69%)
		No = 38(31%)
18.	Postoperative Complications	
	I. Diabetes Insipidus	Yes = 55 (40%)
		No = 82 (59.85%)
	II. Hyponatremia	Yes = 34 (24.8%)
		No = 103 (75.18%)
	III. Leakage of CSF	Yes = 22 (16%)
		No = 115 (84%)
	IV. Cranial Nerve Palsy	Yes = 17(12%)
		No = 120 (88%)
	V. Hypocortisolism	Yes = 73 (53%)
		No = 64 (47%)
	VI. Hypothyroid	Yes = 78 (57%)
		No = 59 (43%)
	VII. Sex Hormone Replacement	Yes = 37(27%)
		No = 100 (73%)
19.	Postoperative Mortality (within 8 weeks)	Yes = 2 (1%)
		No = 135 (99%)
20.	Residual Tumor	Yes = 58 (43%)
		No = 77 (57%)
21.	Tumor Recurrence/Regrowth	Yes = 17 (22%)
		No = 60 (78%)
22.	First Repeat Surgery	Yes = 29 (39%)
		No = 46 (61%)
23.	Second Repeat Surgery	Yes = 9 (31%)
		No = 20 (69%)
24.	External Radiation Therapy	Yes = 13 (17%)
		No = 62(83%)
25.	Cyber/Gamma Knife	Yes = 10 (16%)
		No = 52(84%)

## Discussion

NFPAs are complex in their presentation and management outcomes in our region, and more NFPAs were observed in males with a mean age of 48 years at initial clinical presentation. Our data regarding gender and age distribution at the time of initial presentation are consistent with a recent study in an Irani population [10]. Most of our study participants presented with visual field defects (77%) and headache (55%), consistent with most of the published series to date [11].

Pituitary hormone deficiency at the time of presentation was less common than what has been reported in some other studies [12,13]. Only 11% of our total study participants presented with symptoms of hypothyroid, 6% with symptoms of hypocortisolism and gonadotrophic deficiency, and only 1% had symptoms of growth hormone deficiency. Pituitary apoplexy was the presenting feature in 10% of our patients, a prevalence near that reported in other series [14].

Two-thirds of our patients had macroadenoma on first MRI scan, a slightly larger portion than that reported in the literature. It may be because of delay in health-seeking and poverty; as people with fewer resources may ignore their usual symptoms like headache and may not seek timely medical attention. Another factor contributing to delayed proper care is the unavailability of specialized healthcare like endocrine, neurosurgery and radiology setups in many cities of our country.

We observed decreased pituitary function after TSS, similar to a report from Korea [15]. However, the overall literature regarding the recovery of the hypothalamic-pituitary axis function after surgical resection of NFPAs is conflicting [16,17]. Several studies report a variable degree of improvement in pituitary gland function, whereas other studies could not demonstrate significant improvement in the function of the pituitary gland. We observed transient DI in 31% of the population, which is similar to that reported in the literature 20% to 30% [18]. Permanent diabetes insipidus was seen in 9%, which is a higher incidence compared to previous reports (up to 5%) [19]. This decrease in the pituitary function and higher incidence of DI in our study may be due to the large tumor size and presentation with compressive symptoms which may have cumulatively resulted in aggressive tumor resections.

The success of tumor resection surgery varies and depends directly on the experience and skills of the surgeon and inversely with the diameter and consistency of the tumor, its adhesion, and invasion [17,20]. The total tumor resection rate in the literature is highly variable from 20% to 83% with mean approximately 50% of patients having residual disease after surgery, as also observed in our study (43%) [9]. The reason for this high residual disease and decreased pituitary function after TSS in our study could be because of the large size of the tumor and the male majority of our study population as these two factors have been reported to be associated with high residual burden [21].

The recurrence/regrowth of the adenoma in our study was 22%, which falls within the reported 10% to 40% recurrence rate in other studies, depending on whether adjunctive radiotherapy was used or not [22,23].

TSS remained the treatment of choice in most study centers, especially in patients with visual symptoms and apoplexy because, through this treatment modality, immediate decompression of the optical pathways can be done. Improvement in vision has been observed in up to 80% of patients, which is slightly higher than what we found in our study (69%) [24].

Our results align with previous studies that indicate tumor resection through TSS has very low mortality and a low rate of major complications when performed by skilled neurosurgeons [25].

This was the first clinical report on NFPAs from a Pakistani population on patient presentation, management, and outcomes after surgery. Findings from this study allow for a comparison of the differences in management and outcomes of NFPAs with the other countries in this region due to variations in the availability of resources. Our study has some limitations due to the retrospective nature of the data collection, and in that, it was a single-center patient cohort and, therefore, not a true representation of the entire population. Also, serum antidiuretic hormone and immunohistochemical staining of surgical specimens were not available in our institute, which would have given a robust platform to analyze the study further.

## Conclusions

In Pakistan, patients with NFPAs are more likely to present during a later stage with larger adenomas and compressive symptoms compared to patients in developed countries. TSS, if well planned, can be safe and efficacious for most patients with NFPAs, even in developing countries. For the detection of residual disease and tumor recurrence, close screening and a multidisciplinary approach are needed in the post-surgery period.
